# Supplementary material for: A nonS-locus F-box gene breaks self-incompatibility in diploid potatoes
Source: Nat Commun. 2021 Jul 6;12:4142. doi: 10.1038/s41467-021-24266-7 (PMC8260799; doi:10.1038/s41467-021-24266-7)
Supplement: Supplementary file 1 — Supplementary Information [file 41467_2021_24266_MOESM1_ESM.pdf]

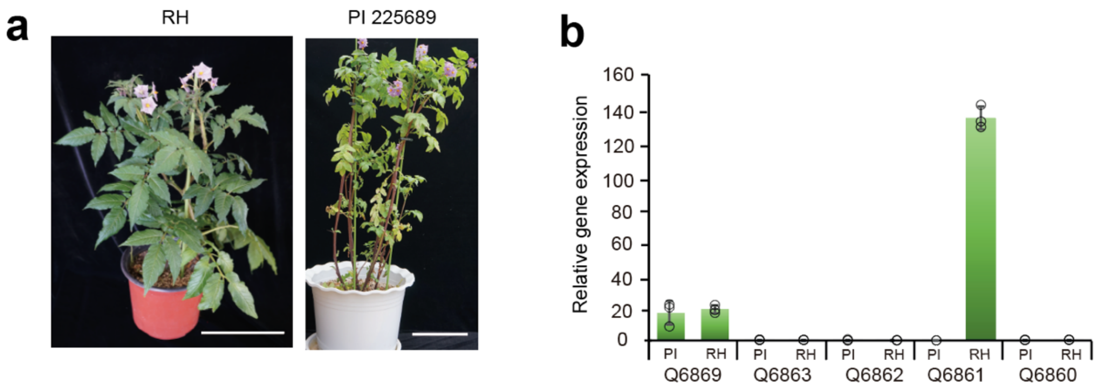

Supplementary Fig. 1 | Growth status of two parent lines and expression pattern of the candidate genes in the two parent lines.

a, Status of two parent lines used in mapping the *NSF* gene. Bar is 10 cm. **b,** Expression of the five annotated genes in the pollen of RH and PI 225689, normalized against an endogenous reference gene (*StEF1-a*, elongation factor 1α). Data are presented as means ± s. d. (n = 3 biological replicates). PI, PI 225689; 6869, PGSC0003DMG400016869; 6863, PGSC0003DMG400016863; 6862, PGSC0003DMG400016862; 6861, PGSC0003DMG400016861; 6860, PGSC0003DMG400016860.

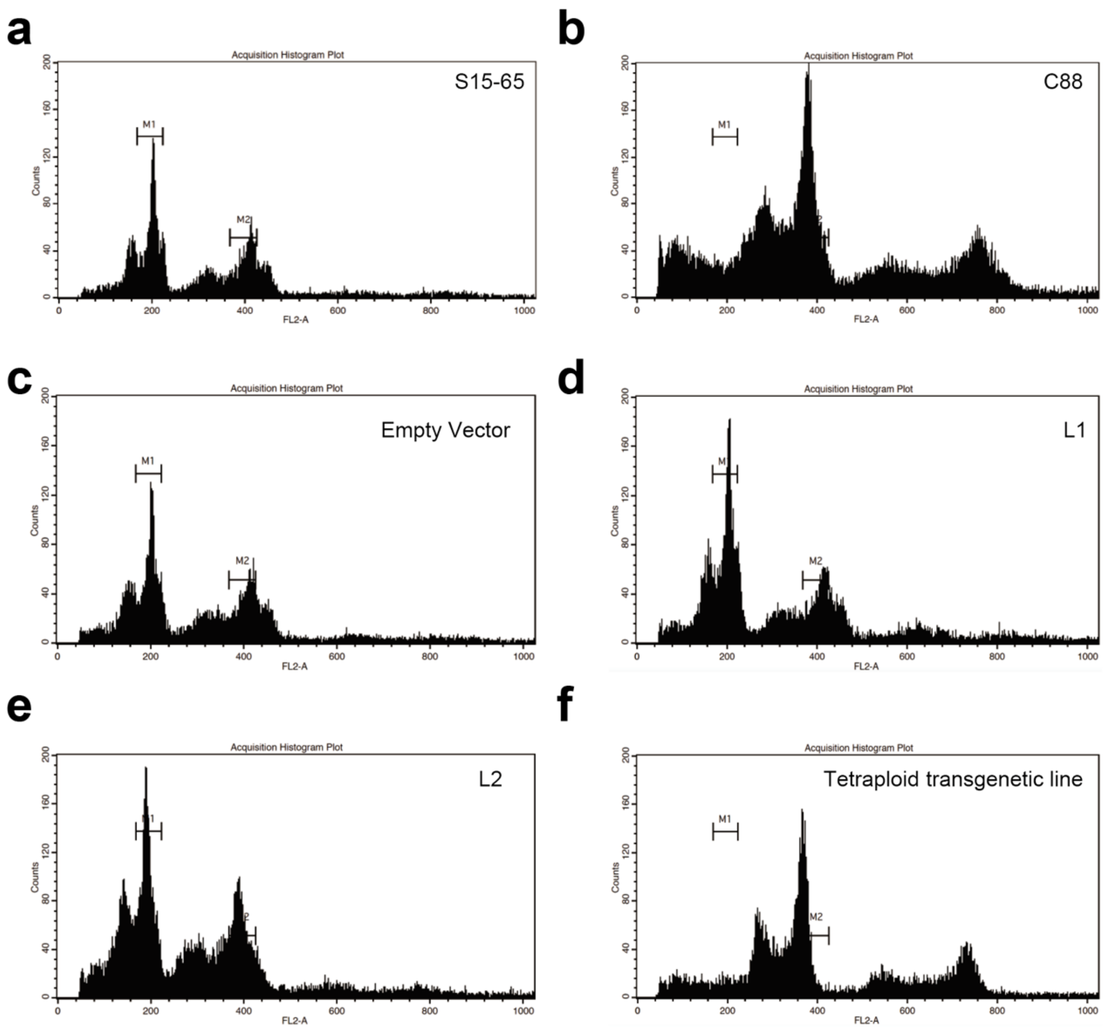

Supplementary Fig. 2 | Flow cytometry analysis of the *NSF* transgenic lines. Flow cytometry result of a diploid line (**a**), a tetraploid line (**b**), the plant transformed with an empty vector (**c**), the two *NSF* transgenic lines L1 (**d**) and L2 (**e**), and the tetraploid *NSF* transgenic line (**f**). M1 and M2 are two representative peaks detected only in the diploid lines, whereas other peaks in addition to M1 and M2 present in the tetraploid lines. S15-65, a diploid potato clone from *S. tuberosum* group Phureja; C88, corporation 88, a popular tetraploid cultivar in China.

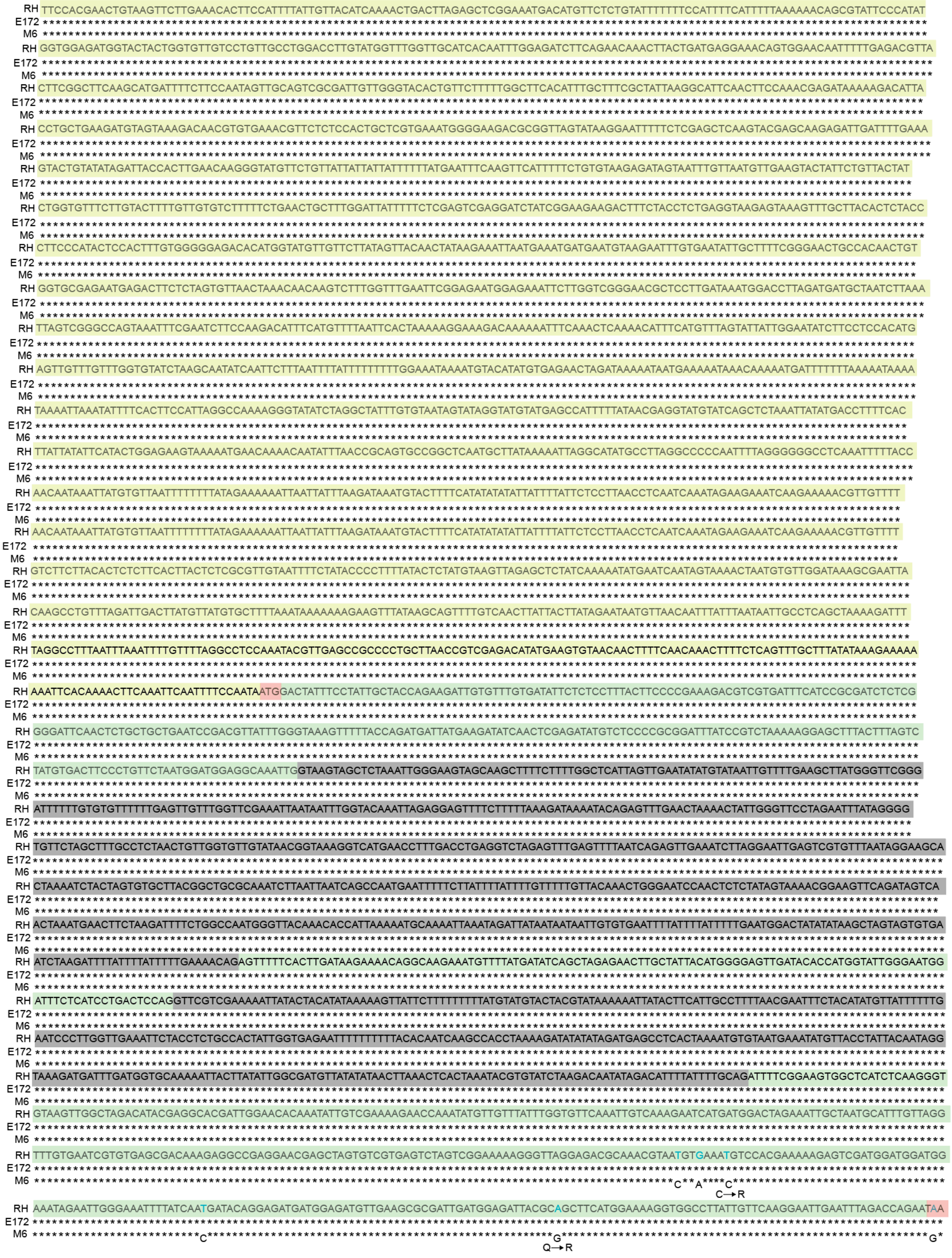

Supplementary Fig. 3 | DNA alignment of the *NSF/Sli* cloned from three SC lines (*S. chacoense* Bitt., M6, and RH). Promoter region (2100 bp) is highlighted in yellow, while exon and intron regions are highlighted in green and gray, respectively. The start and stop codon are highlighted in red. Five DNA base changes were observed in the coding region between RH and M6, two of which lead to amino acid change in protein sequence.

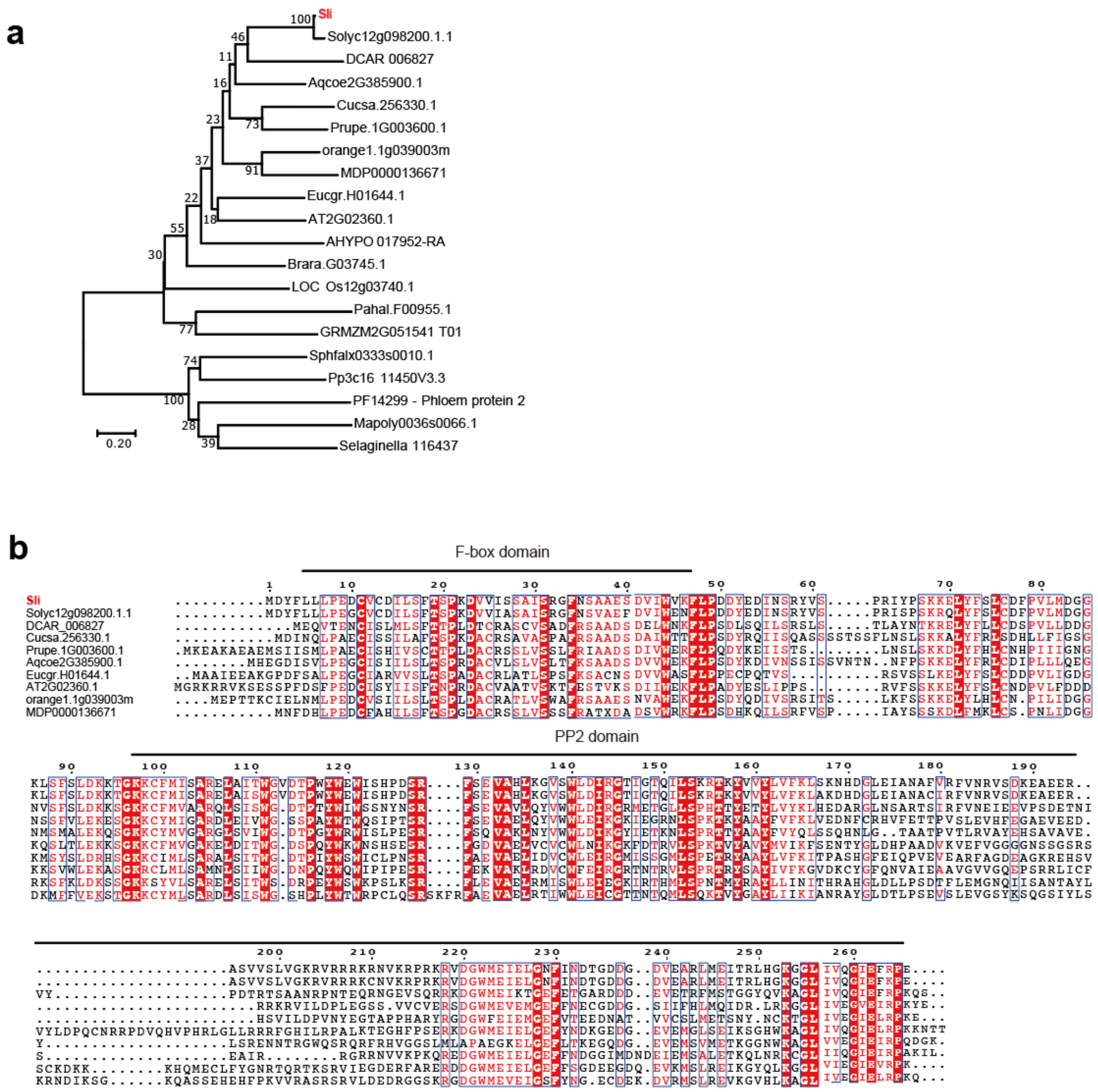

Supplementary Fig. 4 | Phylogenetic analysis and protein alignment of Sli.

a, Phylogenetic tree of Sli (indicated in red) and homologous F-box proteins identified in other plants. The tree was generated with protein sequences by the maximum-likelihood method, with 1,000 bootstraps. **b**, Protein sequence alignment between Sli and F-box proteins identified in other plants. Two conserved domains, F-box and PP2, were identified in Sli.

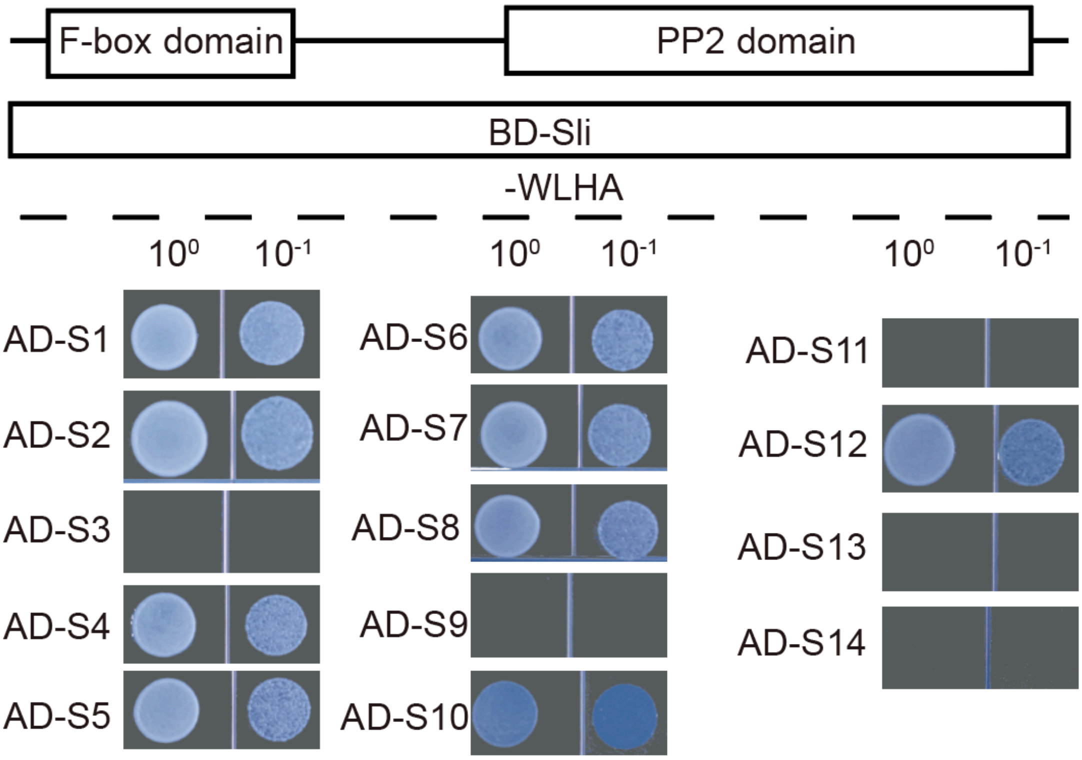

Supplementary Fig. 5 | Y2H analysis of the interactions between full length Sli and 14 S-RNases.

The conserved domains of Sli are indicated by rectangles. The interactions between the 14 potential S-RNases and the PP2 domain of Sli were shown in Fig. 3b. WLHA, synthetic dropout media lacking tryptophan, leucine, histidine and adenine. BD, binding domain; AD, activation domain.

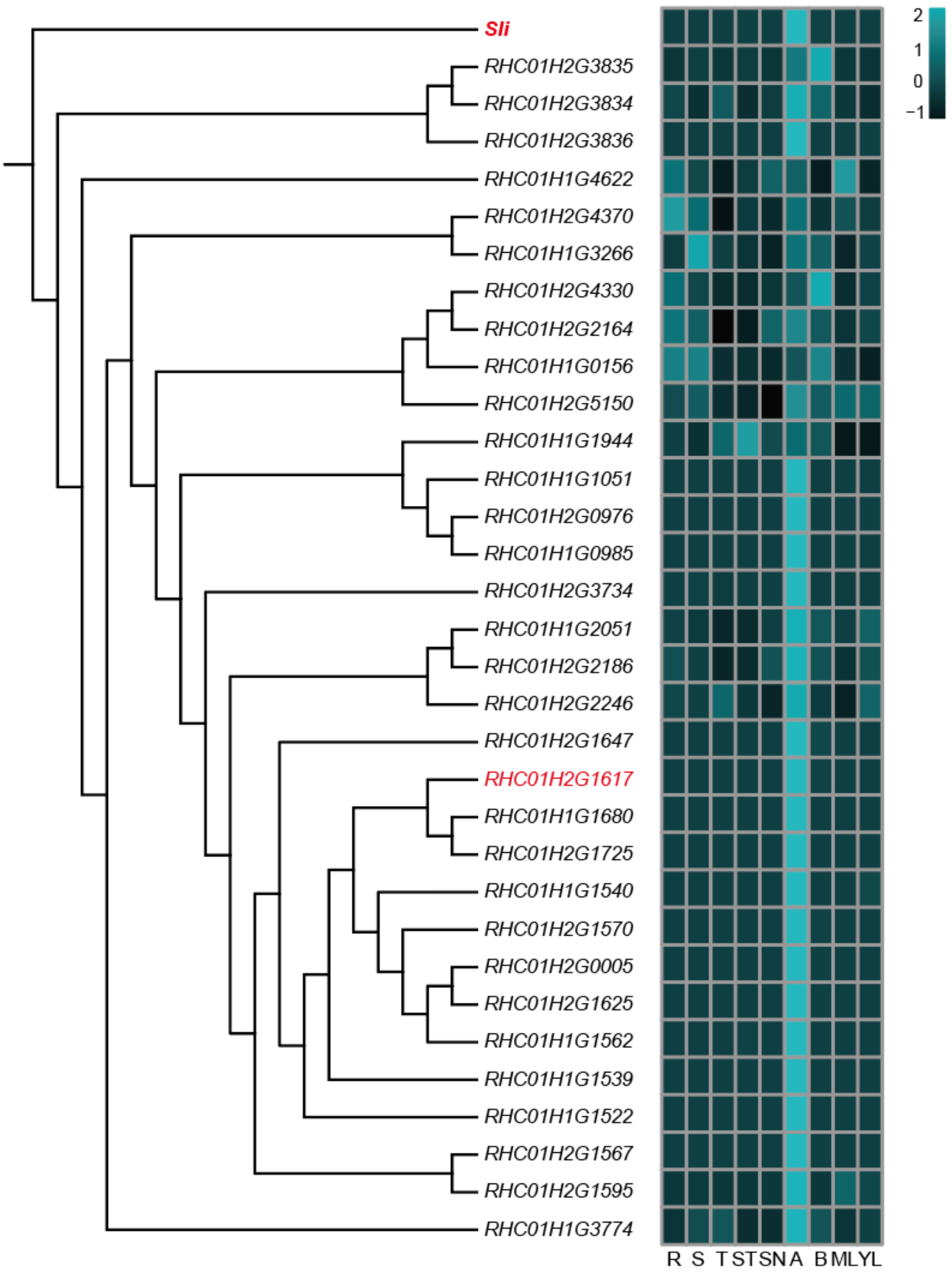

Supplementary Fig. 6 | Phylogenic tree analysis of potential SLFs in RH genome

The annotated *F-box* genes locating within chromosome 1 and highly expressed in the stamen of RH are considered as the potential *SLFs*. Phylogenetic tree of *Sli* (indicated in red) and these *SLFs* was generated using the maximum-likelihood method with 1,000 bootstraps. The *SLF* gene closed to the S-RNase in RH was selected as the negative gene (indicated in red), and was used in the Y2H assay. The expression profiles of these *F-box* genes in RH are illustrated by a gradient in cyan. Z-scores were calculated using the average FPKM value of each gene in different tissues. The heatmaps were plotted base on the z-score to represent the expression pattern. R, root. S, stem. T, tuber. ST, swelling tuber. SN, stolon. A, anther. B, bud. ML, mature leaf. YL, young leaf.

Supplementary Table 1. Self-pollinations of F_1 hybrids derived from the cross between E172 or RH and self-incompatible lines

Accession Number	PG Number	Species[1]	S-RNase Type[2]	Phenotyping of F_1 hybrids[3]
		pollen donor (E172)		
CIP 700235	PG6055	*S.stn*	S10/S13	Y
CIP 700407	PG6059	*S.stn*	S1/S11	Y
CIP 701165	PG6063	*S.stn*	U	Y
CIP 701570	PG6219	*S.phr*	S7/S9	Y
CIP 701611	PG6135	*S.gnc*	S13/?	Y
CIP 701882	PG6137	*S.gnc*	U	Y
CIP 701947	PG6066	*S.stn*	S3/?	Y
CIP 702249	PG6138	*S.gnc*	S6/S7	Y
CIP 702286	PG6024	*S.stn*	U	Y
CIP 702353	PG6071	*S.stn*	S9/?	Y
CIP 702467	PG6139	*S.gnc*	U	Y
CIP 702588	PG6026	*S.stn*	S9/?	Y
CIP 702610	PG6027	*S.stn*	U	Y
CIP 702815	PG6073	*S.stn*	S3/?	Y
CIP 702961	PG6140	*S.gnc*	U	Y
CIP 703034	PG6141	*S.gnc*	U	Y
CIP 703168	PG6142	*S.gnc*	S9/S12	Y
CIP 703197	PG6075	*S.stn*	U	Y
CIP 703244	PG6133	*S.gnc*	S11/?	Y
CIP 703275	PG6143	*S.gnc*	S11/?	Y
CIP 703279	PG6134	*S.gnc*	S10/S13	Y
CIP 703280	PG6144	*S.gnc*	U	Y
CIP 703282	-	*S.gnc*	U	Y
CIP 703294	-	*S.phr*	U	Y
CIP 703308	PG6220	*S.phr*	S3/?	Y
CIP 703312	PG6079	*S.stn*	S3/?	Y
CIP 703315	PG6146	*S.gnc*	U	Y
CIP 703317	PG6080	*S.stn*	S8/?	Y
CIP 703352	PG6147	*S.gnc*	S10/?	Y
CIP 703446	PG6083	*S.stn*	S3/?	Y
CIP 703473	PG6029	*S.stn*	U	Y
CIP 703514	-	*S.phr*	S6/?	Y
CIP 703539	PG6207	*S.phr*	U	Y
CIP 703541	-	*S.phr*	S2/S9	Y
CIP 703545	PG6208	*S.phr*	S3/S7	Y
CIP 703579	-	*S.phr*	U	Y
CIP 703580	-	*S.phr*	U	Y
CIP 703581	-	*S.phr*	S1/S11	Y
CIP 703595	-	*S.phr*	S1/S6	Y
CIP 703637	PG6084	*S.stn*	S3/?	Y
CIP 703654	PG6221	*S.phr*	U	Y
CIP 703698	PG6085	*S.stn*	U	Y
CIP 703767	PG6222	*S.phr*	S6/?	Y

CIP 703774	PG6030	*S.stn*	U	Y
CIP 703783	PG6087	*S.stn*	S3/S11	Y
CIP 703800	PG6223	*S.phr*	S11/?	Y
CIP 703812	PG6224	*S.phr*	U	Y
CIP 703823	PG6093	*S.stn*	U	Y
CIP 703825	PG6148	*S.gnc*	U	Y
CIP 703831	PG6150	*S.gnc*	U	Y
CIP 703836	PG6151	*S.gnc*	U	Y
CIP 703870	PG6152	*S.gnc*	U	Y
CIP 703959	PG6096	*S.stn*	S9/?	Y
CIP 703998	PG6099	*S.stn*	S9/?	Y
CIP 704022	PG6101	*S.stn*	S3/?	Y
CIP 704043	PG6102	*S.stn*	S9/?	Y
CIP 704254	PG6153	*S.gnc*	U	Y
CIP 704393	PG6155	*S.gnc*	S3/S8	Y
CIP 704481	PG6156	*S.gnc*	S10/?	Y
CIP 704490	PG6157	*S.gnc*	U	Y
CIP 704571	PG6104	*S.stn*	S6/?	Y
CIP 704797	PG6036	*S.stn*	S9/S11	Y
CIP 704823	PG6041	*S.stn*	S1/?	Y
CIP 704848	PG6044	*S.stn*	S3/?	Y
CIP 704856	PG6045	*S.stn*	S11/?	Y
CIP 704950	PG6050	*S.stn*	U	Y
CIP 704969	PG6023	*S.stn*	S11/?	Y
CIP 705458	PG6158	*S.gnc*	U	Y
CIP 705468	PG6159	*S.gnc*	S8/S10	Y
CIP 705477	PG6106	*S.stn*	S5/S10	Y
CIP 705478	PG6160	*S.gnc*	S2/S7	Y
CIP 705489	PG6109	*S.stn*	S11/?	Y
CIP 705534	PG6111	*S.stn*	S3/?	Y
CIP 705586	PG6117	*S.stn*	U	Y
CIP 705638	PG6119	*S.stn*	S10/?	Y
CIP 705804	-	*S.phr*	S2/S11	Y
CIP 705965	PG6164	*S.gnc*	S8/?	Y
CIP 706205	PG6006	*S.ajh*	U	Y
CIP 706883	PG6124	*S.stn*	U	Y
CIP 706910	PG6125	*S.stn*	S3/?	Y
CIP 707200	PG6126	*S.stn*	S2/?	Y
PI 225689	-	*tbr tbr*	S6/?	Y
		pollen donor (RH)		
CIP 701165	PG6063	*S.stn*	U	Y
CIP 702287	PG6025	*S.stn*	S9/S12	Y
CIP 702815	PG6073	*S.stn*	S3/S9	Y
CIP 702961	PG6140	*S.gnc*	S9/?	Y
CIP 703034	PG6141	*S.gnc*	U	Y
CIP 703352	PG6147	*S.gnc*	S9/S10	Y
CIP 703572	-	*S.phr*	S6/S7	Y

CIP 703654	PG6221	*S.phr*	U	Y
CIP 703831	PG6150	*S.gnc*	S1/S9	Y
CIP 704120	PG6225	*S.phr*	S1/S6	Y
CIP 704254	PG6153	*S.gnc*	U	Y
CIP 705478	PG6160	*S.gnc*	S2/S7	Y
CIP 705500	PG6161	*S.gnc*	S12/?	Y
CIP 705500	PG6161	*S.gnc*	S10/?	Y
PI 197762	-	*tbr tbr*	S6/S11	Y
PI 225671	-	*tbr tbr*	S6/S9	Y
PI 225689	-	*tbr tbr*	S6/?	Y
PI 283119	-	*tbr tbr*	S2/?	Y
PI 310490	-	*tbr tbr*	S6/?	Y
-	-	*S.phr*	S9/S11	Y
PI 604208	-	*S.ctl*	S8/S12	Y
PI 611078	-	*tbr adg*	U	Y
PI 611078	-	*tbr adg*	U	Y
PI 664476	-	*tbr adg*	U	Y
GS 217	-	*tbr tbr*	S2/?	Y
PI 283127	PG6189	*tbr adg*	S2/?	Y
PI 230513	PG6012	*S.stn*	S8/S9	Y
PI 234010	PG6015	*S.stn*	S9/?	Y
		pollen donor (E172)		
CIP 700313	PG6056	*S.stn*	S2/?	U
CIP 702142	PG6069	*S.stn*	S11/?	U
CIP 702287	PG6025	*S.stn*	S12/?	U
CIP 703421	PG6028	*S.stn*	U	U
CIP 703510	PG6206	*S.phr*	U	U
CIP 703548	PG6210	*S.phr*	S1/S9	U
CIP 703570	-	*S.phr*	S2/S11	U
CIP 704120	PG6225	*S.phr*	S1/S6	U
CIP 704270	PG6154	*S.gnc*	S2/?	U
CIP 705079	-	*S.phr*	U	U
CIP 705476	PG6105	*S.stn*	U	U
CIP 705479	PG6107	*S.stn*	S11/?	U
CIP 705553	PG6113	*S.stn*	S2/S3	U
CIP 705934	PG6163	*S.gnc*	S10/?	U
CIP 706116	PG6165	*S.gnc*	S12/?	U

[1] *S.stn* : *Solanum stenotomum* ; *S.gnc* : *Solanum goniocalys* ; *S.phr* : *Solanum phureja* ; *S.ajh* : *Solanum ajanhuiri* ; *tbr tbr* : *Solanum tuberosum* subsp. *tuberosum* L. ; *tbr adg* : *Solanum tuberosum* subsp. *andigena* H.; *S.ctl* : *Solanum curtilobum*

[2] determinted by PCR and resequencing; U: uncertain, more than two bands or no band of PCR result, suggesting the S-RNase types can not determinated by PCR; ?: only one band of PCR product, suggesting only one S-RNase type can be determinated and the other type is unknown.

[3]whether there are SC individuals in F_1 population or not:Y means 'Yes'; U is abbreviated for 'Uncertain'

Supplementary Table 2. Primers used in this study

Primers for gene mapping

Indel Markers	Sequences
M-1	ACATCTTTTGGCCGTACAT<br>GAGGAAACACTAGTTGGTGG
M-2	AGAAGGGGCAGATTATTAGC<br>TGTAATTGAAGCTCACCTTG
C7-1	TGAATTCATTGCCTACACAG<br>CCTTGACACCTCATCAATTT
D5-2	AATTAACTCCGATTTACCGC<br>ACTTGCTTCAACAGGCTAAG
F3-4	AAGAAGGAATACCCAACTGC<br>GAACTATGTTTCCCCAACTG
F1-4	AATCAGGTCTACTCCCCTGT<br>AACCTGGTCAACAAGAAAGA
F2-3	GATGCTATAAAACAGGTGCC<br>AATCCTTCTAAATATGGCCC

Primers for qRT-PCRassays

Target Gene	Sequences
Q6869	CGGTGAGAAGATTGAGCTTCTAGTCGA<br>CCGCATAGACGGATGGAACCTATG
Q6863	GTCTATGTGACTTCCCTGTTCTAATGGATG<br>CATGGTGTATCAACTCCCCATGAAATAG
Q6862	AGAGTGATACTGAGACTGAAGAACAAGCCA<br>GGACCATCACTACCTTCCTTGCTATTGA
Q6861	GTCTATGTGACTTCCCTGTTCTAATGGATG<br>CATGGTGTATCAACTCCCCATGAAATAG
Q6860	GATTTGAATGGACGGTAGCAAAGTT<br>CCATATGGGAATACGAGTTCGTGGAA
QEF1-α	GATGGTCAGACCCGTGAACA<br>CCTTGGAGTACTTCGGGGTG

Primers for the gene transformation

com-6861-F	cggtacccggggatc AAGACATTACCTGCTGAAGATGTAGTAAAGAC
com-6861-R	ggccagtgccaagct GTGTGCCATACTTTCGAGACAACTCCT

Primers for *In situ* hybridaziton

Prob-Sli-F	ATGGAGGCAAATTGAGTTTTTCACTTGAT
Prob-Sli-R	taatacgactcactatagg TTTGACAATTTGAACACCAAATAAACAACATATT

Primers for the determination of S-RNase types

S-RNase types	Sequences

S-RNase1	AGTCCGAACCACAAGATGTTT<br>TGAAGAGTTTATTTATCATCGGAAC
S-RNase2	GGGGAAACTGGAAAATGGTT<br>ATGTGAAGTTGTTCAGCGAAA
S-RNase3	ATGTTTAAATCACTGCTTACATC<br>TAGAAGTTGTTCAGGGACGG
S-RNase4	CGAAGAAGAATAGATGAGAACG<br>CAAGATGCGTAACTACCC
S-RNase5	ACTGGTAGTTGTGTAGTGTGGA<br>GCTAGGAACATAGATGACAGAGAT
S-RNase6	ACACCCCAGCAGAAAGAAAA<br>GCCGCATAAATAAAGCATCGG
S-RNase7	GGGAAGGAAACGAAAAGGTGT<br>GCCTGGAGAGTTTGCATTTAAA
S-RNase8	CGGAAGAAAGGAAATGAAGTGAG<br>AATTACAGCAAGGGGAGGGG
S-RNase9	CAACAAAATGGCTAAATCGCAG<br>GGTTTTCTGTTGGGTGGCAT
S-RNase10	ACACCCTTAGTCACCGATGG<br>TTCTGCATGTAGGTTCTGCA
S-RNase11	CTCAAGTGACCAGTAGAGGG<br>CCAACCTGGTCATTCAAAAT
S-RNase12	ACCAGAAACACCATAAGTCG<br>ATGAGAAACAAAACCTACGG
S-RNase13	CACAGAGTGTCTAAGTCAG<br>CTAAACCTATTGTCTGGTAACG
S-RNase14	GCTCATGCCAGTATTCTT<br>TATGTCACCAGGATCAGAAT

**Primers for Y2H assay**	
BD-Sli-F	ggcggtacc ATGGACTATTTCCTATTGCTACCAGAAGGT
BD-Sli-R	ggcggatcc TCATTCTGGTCTAAATTCAATTCCTTGAAC
BD-PP2-F	ggcggtacc GGCAAATTGAGCTTTTCACTTGATAAGA
BD-PP2-R	ggcggatcc TCATTCTGGTCTAAATTCAATTCCTTGAAC
BD-1617-F	ggcgaattc CAACAGCTCCTTGGTTCTTCTCATG
BD-1617-R	ggcggatcc AACTTGTGTAGCTTCGTTGCTTTCTC
AD-S1-F	ggccatatg ATGGGGGATTTCGAGTTATTG
AD-S1-R	ggcgagctc TCGGAACTTTATCCTAGG
AD-S2-F	ggccatatg ATGGGGGATTTCGATTCCCTCC
AD-S2-R	ggcgagctc GCGAAAAAAGATTTTTCCC
AD-S3-F	ggccatatg ATGGCGGATTTCGACAAATTGC
AD-S3_R	ggcgagctc GGGACGGAAAAATATTTTCCC
AD-S4-F	ggccatatg ATGGGGGATTTTGATTACATGC
AD-S4-R	ggcgagctc TCTAAATAAAATCGCCGT
AD-S5-F	ggccatatg ATGTCTGAATTAATTACTAAACC
AD-S5-R	ggcgagctc GTTTGGAAATATGAGATT
AD-S6-F	ggccatatg ATGGGTCATTTCGAGTATTTCC
AD-S6-R	ggcgagctc TCGAAAAAAAATCCTTTCC

AD-S7-F	ggccatatg ATGGGGGATTTCGAGTTATTGG
AD-S7-R	ggcgagctc TCGGAACTTTATTTTATG
AD-S8-F	ggccatatg ATGGGGACTTTTGACCAATTGC
AD-S8-R	ggcgagctc TGGAAATGCAATCAGAT
AD-S9-F	ggccatatg ATGGGGGATTTCGACTATATGC
AD-S9-R	ggcgagctc TAATCGAAACAAAATCAGCGG
AD-S10-F	ggccatatg ATGGGGAATTTCGATCAATTGC
AD-S10-R	ggcgagctc TCTACGAAAAAAGATTTC
AD-S11-F	ggccatatg ATGGGGGATTTCGATTCATTGC
AD-S11-R	ggcgagctc ACTACGGAAAAATATGTC
AD-S12-F	ggccatatg ATGGGAGATTTCGATTCATTGC
AD-S12-R	ggcgagctc GCTTCGAAAAAAGATTTC
AD-S13-F	ggccatatg ATGGGGAATTTCGACTACCTGC
AD-S13-R	ggcgagctc TCGAAACAAAATACTCAT
AD-S14-F	ggccatatg ATGGCGAATTTCGACTACATGC
AD-S14-R	ggcgagctc ATTTGGAATTTCAATCACC

**Primers for Luciferase complementation assay**	
cLUC-PP2-F	ggcggtacc ATGGTAAAGTTTTACCAGATGATTATGAAGATA
cLUC-PP2-R	ggcggatcc TCATTCTGGTCTAAATTCAATTCCTTGAAC
nLUC-S1-F	gagctcggtacccgggatcc ATGGGGGATTTCGAGTTATTG
nLUC-S1-R	gcgtacgagatctggtcgac TCGGAACTTTATCCTAGG
nLUC-S2-F	gagctcggtacccgggatcc ATGGGGGATTTCGATTCCCTCC
nLUC-S2-R	gcgtacgagatctggtcgac GCGAAAAAAGATTTTTCCC
nLUC-S3-F	gagctcggtacccgggatcc ATGGCGGATTTCGACAAATTGC
nLUC-S3-R	gcgtacgagatctggtcgac GGGACGGAAAAATATTTTCCC
nLUC-S4-F	gagctcggtacccgggatcc ATGGGGGATTTTGATTACATGC
nLUC-S4-R	gcgtacgagatctggtcgac TCTAAATAAAATCGCCGT
nLUC-S5-F	gagctcggtacccgggatcc ATGTCTGAATTAATTACTAAACC
nLUC-S5-R	gcgtacgagatctggtcgac GTTTGGAAATATGAGATT
nLUC-S6-F	gagctcggtacccgggatcc ATGGGTCATTTCGAGTATTTCC
nLUC-S6-R	gcgtacgagatctggtcgac TCGAAAAAAAATCCTTTCC
nLUC-S7-F	gagctcggtacccgggatcc GGGGATTTCGAGTTATTGG
nLUC-S7-R	gcgtacgagatctggtcgac TCGGAACTTTATTTTATG
nLUC-S8-F	gagctcggtacccgggatcc ATGGGGACTTTTGACCAATTGC
nLUC-S8-R	gcgtacgagatctggtcgac TGGAAATGCAATCAGAT
nLUC-S9-F	gagctcggtacccgggatcc ATGGGGGATTTCGACTATATGC
nLUC-S9-R	gcgtacgagatctggtcgac TAATCGAAACAAAATCAGCGG
nLUC-S10-F	gagctcggtacccgggatcc ATGGGGAATTTCGATCAATTGC
nLUC-S10-R	gcgtacgagatctggtcgac TCTACGAAAAAAGATTTC
nLUC-S11-F	gagctcggtacccgggatcc ATGGGGGATTTCGATTCATTGC
nLUC-S11-R	gcgtacgagatctggtcgac ACTACGGAAAAATATGTC
nLUC-S12-F	gagctcggtacccgggatcc ATGGGAGATTTCGATTCATTGC
nLUC-S12-R	gcgtacgagatctggtcgac GCTTCGAAAAAAGATTTC
nLUC-S13-F	gagctcggtacccgggatcc ATGGGGAATTTCGACTACCTGC
nLUC-S13-R	gcgtacgagatctggtcgac TCGAAACAAAATACTCAT
nLUC-S14-F	gagctcggtacccgggatcc ATGGCGAATTTCGACTACATGC
nLUC-S14-R	gcgtacgagatctggtcgac ATTTGGAATTTCAATCACC

Supplementary Table 3. Accession numbers of *S-RNase* related genes used in phylogenetic analysis

Gene Name	Accession Number
Ss10	AOA60123.1
Ss9	AOA60122.1
Ss8	AOA60121.1
Ss7	AOA60120.1
Ss6	AOA60119.1
Ss5	AOA60118.1
Ss4	AOA60117.1
Ss3	AOA60116.1
Ss2	AOA60115.1
Ss1	AOA60114.1
So5	AOA60111.1
So4	AOA60110.1
So3	AOA60109.1
So2	AOA60108.1
So1	AOA60107.1
S18	AOY36484.1
S17	AOY36483.1
S23	AOY36489.1
S22	AOY36488.1
S24	APM86803.1
S14	AAF36980.1
S11	AAB30528.1
S2-protein	CAA40216.1
S3-protein	CAA40217.1
S-arg S16	AAY26141.1
Sp2	AOA60113.1
Sp1	AOA60112.1
60_E	AEN02429.1
60_D	AEN02428.1
60_C	AEN02427.1
60_B	AEN02426.1
60_A	AEN02425.1
47_D	AEN02424.1
47_A	AEN02423.1
S-2	CAA44600.1
S-12	AAF05729.1/AAD56217.1
N.ala.NE	AAA21135.1
N.glu. NGR3	BAA84469.1
S.lyc.LE	CAA55895.1
N.glu.NGR2	BAA84468.1
N.tab,NK1	NP_001312837.1
S.lyc,LER	NP_001307144.1
RNase T2	XP_023090050.1
