# Supplementary material for: A nonS-locus F-box gene breaks self-incompatibility in diploid potatoes
Source: Nat Commun. 2021 Jul 6;12:4142. doi: 10.1038/s41467-021-24266-7 (PMC8260799; doi:10.1038/s41467-021-24266-7)
Supplement: Supplementary file 3 — Reporting Summary [file 41467_2021_24266_MOESM3_ESM.pdf]

nature research

Corresponding author(s): shangyi@ynnu.edu.cn

Last updated by author(s): 2021.05.30

Reporting Summary

Nature Research wishes to improve the reproducibility of the work that we publish. This form provides structure for consistency and transparency in reporting. For further information on Nature Research policies, see our Editorial Policies and the Editorial Policy Checklist.

Statistics

For all statistical analyses, confirm that the following items are present in the figure legend, table legend, main text, or Methods section.

n/a	Confirmed	
☐	☒	The exact sample size (n) for each experimental group/condition, given as a discrete number and unit of measurement
☐	☒	A statement on whether measurements were taken from distinct samples or whether the same sample was measured repeatedly
☒	☐	The statistical test(s) used AND whether they are one- or two-sided<br>*Only common tests should be described solely by name; describe more complex techniques in the Methods section.*
☒	☐	A description of all covariates tested
☒	☐	A description of any assumptions or corrections, such as tests of normality and adjustment for multiple comparisons
☐	☒	A full description of the statistical parameters including central tendency (e.g. means) or other basic estimates (e.g. regression coefficient) AND variation (e.g. standard deviation) or associated estimates of uncertainty (e.g. confidence intervals)
☒	☐	For null hypothesis testing, the test statistic (e.g. F, t, r) with confidence intervals, effect sizes, degrees of freedom and P value noted<br>*Give P values as exact values whenever suitable.*
☒	☐	For Bayesian analysis, information on the choice of priors and Markov chain Monte Carlo settings
☒	☐	For hierarchical and complex designs, identification of the appropriate level for tests and full reporting of outcomes
☒	☐	Estimates of effect sizes (e.g. Cohen's d, Pearson's r), indicating how they were calculated

Our web collection on statistics for biologists contains articles on many of the points above.

Software and code

Policy information about availability of computer code

Data collection	The sequencing of four libraries were performed using NovaSeq 6000 platform (Anoroad Gene Technology, Beijing, China)
Data analysis	Platypus (V0.7.4) software, Phytozome v13, Mafft, FACSDiva v7.0

For manuscripts utilizing custom algorithms or software that are central to the research but not yet described in published literature, software must be made available to editors and reviewers. We strongly encourage code deposition in a community repository (e.g. GitHub). See the Nature Research guidelines for submitting code & software for further information.

Data

Policy information about availability of data

All manuscripts must include a data availability statement. This statement should provide the following information, where applicable:
- Accession codes, unique identifiers, or web links for publicly available datasets
- A list of figures that have associated raw data
- A description of any restrictions on data availability

The BSA-Seq data (SRR14637423 and SRR14637422) has been deposited in NCBI under with project number PRJNA732509. All other data that support the findings of this study are available from the corresponding author upon reasonable request.

Field-specific reporting

Life sciences study design

All studies must disclose on these points even when the disclosure is negative.

Sample size	6624 F2 progenies from crossing PI 225689 and RH were used in the genotype assay.
Data exclusions	No data was excluded for analysis.
Replication	This pollination process was repeated for at least 30 times for each clone.
Randomization	This is not relevant to our study. No allocation was involved in this study.
Blinding	Blinding was not relevant to this study, because all data are objective.

Reporting for specific materials, systems and methods

We require information from authors about some types of materials, experimental systems and methods used in many studies. Here, indicate whether each material, system or method listed is relevant to your study. If you are not sure if a list item applies to your research, read the appropriate section before selecting a response.

Materials & experimental systems

n/a	Involved in the study
☒	☐ Antibodies
☒	☐ Eukaryotic cell lines
☒	☐ Palaeontology and archaeology
☒	☐ Animals and other organisms
☒	☐ Human research participants
☒	☐ Clinical data
☒	☐ Dual use research of concern

Methods

n/a	Involved in the study
☒	☐ ChIP-seq
☐	☒ Flow cytometry
☒	☐ MRI-based neuroimaging

Flow Cytometry

Plots

Confirm that:

☒ The axis labels state the marker and fluorochrome used (e.g. CD4-FITC).

☒ The axis scales are clearly visible. Include numbers along axes only for bottom left plot of group (a 'group' is an analysis of identical markers).

☒ All plots are contour plots with outliers or pseudocolor plots.

☒ A numerical value for number of cells or percentage (with statistics) is provided.

Methodology

Sample preparation	The fresh leaves from in vitro plants were chopped and incubated in lysate. Then the cell nucleus were collected by centrifugation.
Instrument	BD LSRFortessa™ X-20
Software	BD FACSDiva v7.0 Software
Cell population abundance	The proportion of target cell was higher than 70%.
Gating strategy	We used known diploid and tetraploid potato clones as control to deduce the ploidy of transgenic plants.

☒ Tick this box to confirm that a figure exemplifying the gating strategy is provided in the Supplementary Information.
