# Supplementary material for: A nonS-locus F-box gene breaks self-incompatibility in diploid potatoes
Source: Nat Commun. 2021 Jul 6;12:4142. doi: 10.1038/s41467-021-24266-7 (PMC8260799; doi:10.1038/s41467-021-24266-7)
Supplement: Supplementary file 4 — Source Data [file 41467_2021_24266_MOESM4_ESM.pdf]

Fig.1c

Individuals	phynotype	markers[1,2]						
		M-1	F1-4	F2-3	C7-1	D5-2	F3-4	M-2
F1-19	SC	A	H	H	H	H	H	H
F1-28	SC	A	A	H	H	H	H	H
F1-29	SI	A	A	A	A	A	H	H
F1-52	SC	H	H	H	H	H	H	A
F1-67	SC	H	H	H	H	H	A	A
F1-98	SC	A	A	H	H	H	H	H
F1-102	SC	A	H	H	H	H	H	H
F1-104	SC	H	H	H	H	H	A	A
F1-136	SI	H	A	A	A	A	A	A
F1-183	SC	A	H	H	H	H	H	H
F1-216	SI	H	A	A	A	A	A	A
F1-233	SC	A	H	H	H	H	H	H
F2-14	/	H	H	H	H	A	A	A
F2-53	/	A	A	A	A	H	H	H

[1]SC genotype=H/B; SI genotype=A; [2] The *NSF* location is indicated in green

Fig.1f

	Replicates	Ct	
		StEF1-α	NSF
PI 225689	1	25.55	Undetermined
	2	25.65	36.65
	3	25.53	37.05
RH	1	25.93	24.31
	2	25.74	24.27
	3	25.68	24.17

Fig.1h

	Replicates	Ct	
		StEF1-α	NSF
Empty Vector	1	25.37	37.02
	2	25.73	37.28
	3	25.58	37.00
L1	1	25.35	24.59
	2	25.20	24.58
	3	25.20	24.58
L2	1	25.60	24.60
	2	25.48	24.66
	3	25.58	24.65

Fig.2c

	Replicates	Ct	
		StEF1-α	NSF
Stage 1	1	25.79	27.37
	2	25.87	27.31
	3	25.82	27.23
Stage 2	1	23.11	24.45
	2	23.12	24.73
	3	23.09	24.35
Stage 3	1	23.52	24.79
	2	23.54	25.03
	3	23.58	24.87
Stage 4	1	23.60	23.73
	2	23.10	25.20
	3	23.27	24.48
Style	1	19.72	22.22
	2	19.57	23.52
	3	19.63	23.87
Pollen	1	25.50	22.35
	2	25.57	21.98
	3	25.43	22.38

Fig. S1

	Replicates	Ct					
		StEF1-α	Q6869	Q6863	Q6862	Q6861	Q6860
PI 225689	1	25.55	28.27	32.46	35.15	Undetermined	33.61
	2	25.65	27.03	32.36	36.65	36.65	32.63
	3	25.53	26.91	34.58	35.22	37.05	33.38
RH	1	25.60	26.90	33.14	34.49	24.31	32.93
	2	25.48	27.11	33.14	33.84	24.27	32.91
	3	25.58	27.26	32.75	36.98	24.17	32.40

FPKM value of the genes used for Fig. S5

Gene ID	root	stem	tuber	swelling tuber	stolon	anther	bud	mature leaf	young leaf
RHC12H2G2976 (NSF)	0.00	0.03	0.00	0.04	0.00	5.49	0.12	0.00	0.00
RHC01H2G3835	0.13	1.03	0.40	0.83	0.17	8.30	14.74	0.29	0.02
RHC01H2G3834	0.79	0.17	1.41	0.08	0.51	4.23	1.75	0.40	0.05
RHC01H2G3836	0.20	0.12	0.28	0.40	0.17	63.08	0.58	0.56	0.88
RHC01H1G4622	5.72	4.56	3.50	4.30	5.23	5.20	3.47	6.75	3.71
RHC01H2G4370	51.47	37.12	12.85	23.46	18.46	39.27	21.05	29.98	24.09
RHC01H1G3266	3.95	7.19	4.00	3.67	3.17	5.60	4.95	3.28	4.10
RHC01H2G4330	39.14	27.44	20.19	21.10	23.37	31.63	56.59	20.95	26.62
RHC01H2G2164	34.68	28.60	4.04	11.48	29.53	39.92	27.16	17.39	21.87
RHC01H1G0156	66.35	68.07	15.00	16.50	11.36	37.23	69.93	15.42	4.89
RHC01H2G5150	3.89	4.58	2.76	2.48	0.94	6.64	4.52	5.10	4.90
RHC01H1G1944	0.06	0.04	0.11	0.17	0.07	0.11	0.08	0.01	0.01
RHC01H1G1051	0.00	0.00	0.00	0.00	0.00	2.07	0.00	0.00	0.00
RHC01H2G0976	0.00	0.04	0.00	0.04	0.00	8.58	0.04	0.00	0.09
RHC01H1G0985	0.00	0.00	0.00	0.00	0.00	4.77	0.02	0.05	0.00
RHC01H2G3734	0.15	0.26	0.08	0.13	0.20	4.24	0.11	0.05	0.09
RHC01H1G2051	2.75	1.99	0.19	0.57	2.33	13.18	4.52	2.39	5.60
RHC01H2G2186	5.24	3.66	0.28	0.94	5.59	17.89	5.60	2.00	5.42
RHC01H2G2246	0.24	0.22	0.42	0.18	0.08	0.79	0.19	0.06	0.40
RHC01H2G1647	0.00	0.00	0.00	0.00	0.00	0.45	0.03	0.00	0.00
RHC01H2G1617	0.00	0.00	0.00	0.00	0.00	1.64	0.04	0.00	0.00
RHC01H1G1680	0.00	0.00	0.00	0.00	0.00	0.41	0.00	0.00	0.00
RHC01H2G1725	0.00	0.00	0.00	0.00	0.00	0.66	0.00	0.00	0.00
RHC01H1G1540	0.00	0.00	0.00	0.00	0.00	0.36	0.00	0.00	0.03
RHC01H2G1570	0.00	0.00	0.00	0.00	0.00	1.47	0.03	0.00	0.00
RHC01H2G0005	0.00	0.00	0.00	0.00	0.00	0.39	0.01	0.00	0.00
RHC01H2G1625	0.00	0.00	0.00	0.00	0.00	0.39	0.01	0.00	0.00
RHC01H1G1562	0.00	0.00	0.00	0.01	0.00	0.42	0.00	0.00	0.00
RHC01H1G1539	0.00	0.00	0.00	0.00	0.00	1.45	0.00	0.00	0.00
RHC01H1G1522	0.00	0.00	0.00	0.00	0.00	1.84	0.00	0.00	0.00
RHC01H2G1567	0.00	0.02	0.00	0.00	0.00	1.07	0.02	0.00	0.00
RHC01H2G1595	0.00	0.01	0.00	0.00	0.00	0.97	0.00	0.34	0.10
RHC01H1G3774	0.02	0.10	0.13	0.00	0.00	0.46	0.12	0.02	0.06
